# Supplementary figures and images for: The PRECISION study protocol: Can cervical stiffness in the second trimester predict preterm birth in high-risk singleton pregnancies? A feasibility, cohort study
Source: PLoS One. 2025 Feb 21;20(2):e0316297. doi: 10.1371/journal.pone.0316297 (PMC11844860; doi:10.1371/journal.pone.0316297)

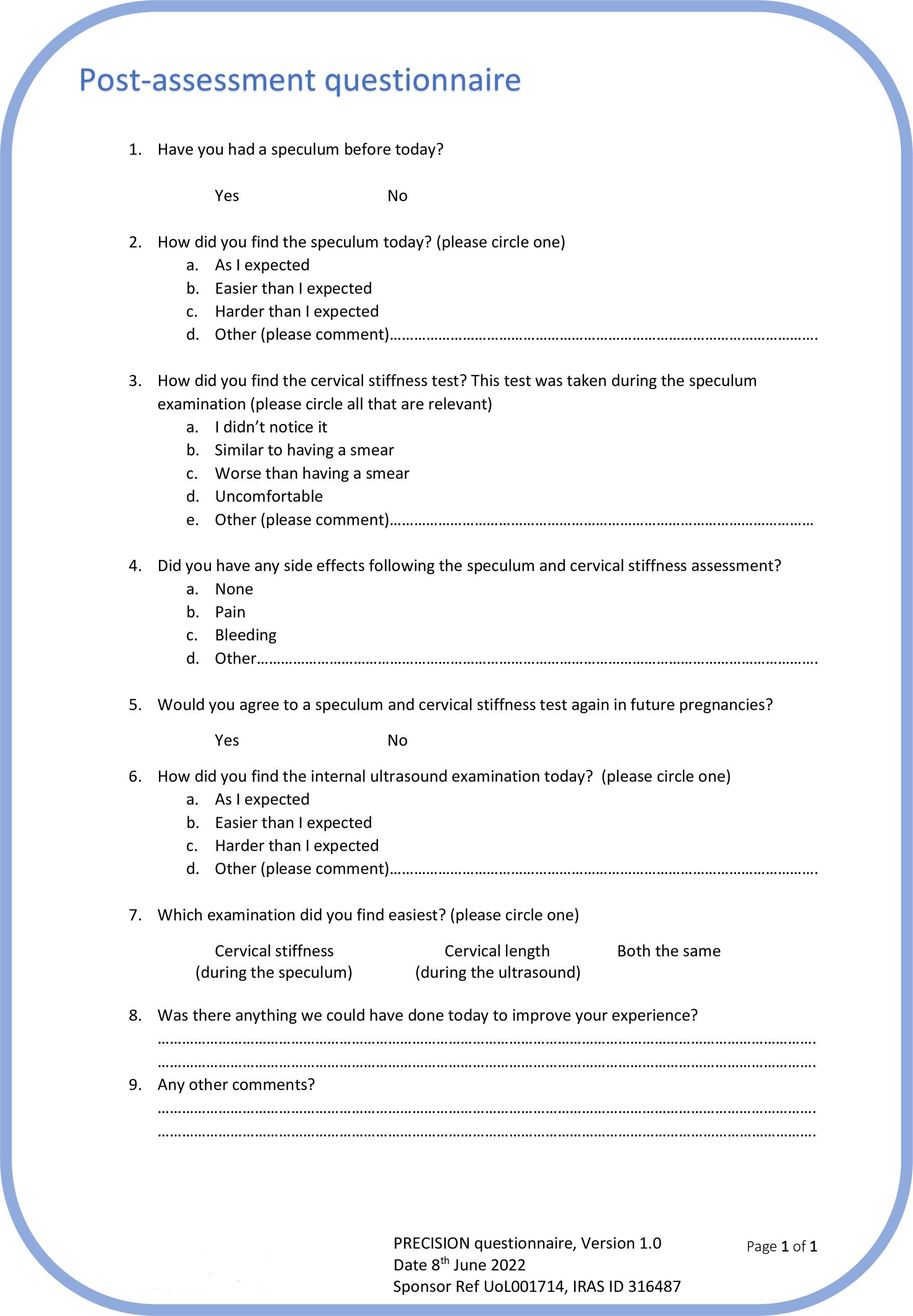

Supplement: S1 Fig — (TIF) [file pone.0316297.s001.tif]
