# Supplementary material for: The PRECISION study protocol: Can cervical stiffness in the second trimester predict preterm birth in high-risk singleton pregnancies? A feasibility, cohort study
Source: PLoS One. 2025 Feb 21;20(2):e0316297. doi: 10.1371/journal.pone.0316297 (PMC11844860; doi:10.1371/journal.pone.0316297)
Supplement: S1 File — (PDF) [file pone.0316297.s002.pdf]

# Participant Consent Form

**PRECISION: Preterm Birth Prediction using Cervical Stiffness.**

Title of Project: Can cervical stiffness in the second trimester predict pre-term birth in high-risk singleton pregnancies?

Name of Researchers:
Dr Elizabeth Medford, Clinical Research Fellow, University of Liverpool/ Liverpool Women's Hospital
Dr Angharad Care, Clinical Lecturer, University of Liverpool/Liverpool Women's Hospital
Dr Andrew Sharp, Senior Clinical Lecturer, University of Liverpool/Liverpool Women's Hospital

Participant Identification Number: ☐☐☐ ☐☐☐

## Please put your <u>initials</u> in each box if you agree with the statement

| | | |
|---|---|---|
| 1. | I confirm that I have read and understand the information sheet dated 23rd February 2023 version 3.2 for the above study. I have had the opportunity to consider the information, ask questions and have had these answered satisfactorily. | ☐ |
| 2. | I understand that my participation is voluntary and that I am free to leave the study at any time, without giving any reason and without my or my baby's medical care or legal rights being affected. | ☐ |
| 3. | I give permission for both my own and my baby's medical notes and data collected during this research to be looked at by responsible individuals involved in this study and regulatory authorities. | ☐ |
| 4. | I give permission for the researcher to contact my medical practitioner in the event of clinically significant findings from this research. | ☐ |
| 5. | I give permission for cervical stiffness measurements to be taken using the Pregnolia device as part of this study. | ☐ |
| 6. | I give permission for vaginal swabs to be taken and used for this study and to be transferred to The University of Liverpool laboratory for analysis and storage during the course of this study. | ☐ |

# Participant Consent Form

| | | |
|---|---|---|
| 7. | I agree for a copy of this completed consent form to be sent to the Department of Women's and Children's Health, Centre for Women's Health Research, University of Liverpool (where it will be kept in a secure location), to allow confirmation that my consent for the study has been given. | ☐ |
| 8. | **OPTIONAL**: I give permission for two additional high vaginal swabs to be taken and transferred to Liverpool Women's Hospital Tissue Bank for analysis and storage for use in future research in other ethically approved research ('gifting'). | **Yes** ☐<br>**No** ☐ |
| 9. | I give permission for my data to be shared both within and outside of the UK in a fully anonymised format. | ☐ |
| 10. | I agree to take part in the above study. | ☐ |

| | | |
|---|---|---|
| | | |
| **Name of participant** | **Signature** | **Date** |

| | | |
|---|---|---|
| | | |
| **Researcher taking consent (if different to PI)** | **Signature** | **Date** |

| | | |
|---|---|---|
| | | |
| **Principal Investigator** | **Signature** | **Date** |

*Original to be kept by the participant; 1 copy for the researcher; 1 copy to be kept with the hospital notes*
