# Supplementary material for: The PRECISION study protocol: Can cervical stiffness in the second trimester predict preterm birth in high-risk singleton pregnancies? A feasibility, cohort study
Source: PLoS One. 2025 Feb 21;20(2):e0316297. doi: 10.1371/journal.pone.0316297 (PMC11844860; doi:10.1371/journal.pone.0316297)
Supplement: S2 File — (PDF) [file pone.0316297.s003.pdf]

# Participant Information Sheet

## PRECISION: Preterm Birth Prediction using Cervical Stiffness.

We are inviting many of the women who attend our pre term birth clinic to take part in a research study. Before you decide whether or not to take part it is important for you to understand why the research is being performed and what it will involve. Please take the time to read the following information carefully and discuss it with others if you wish. One of our team will go through the information sheet with you and answer any questions you may have. Take time to decide whether or not you wish to take part.

**Thank you for reading this.**

**Why are we doing the study?**

The aim of this study is to try find out if there are better ways to test for a woman's risk of having an earlier birth than expected (preterm birth).

We know that preterm birth affects almost 1 in 10 babies in the UK, and sadly preterm birth can be so early that a very small number of babies won't survive. When babies do survive, they can still be at risk of serious harm including damage to the brain, lungs and bowel- all of which can result in life changing disabilities. How severe these problems are is related to how early they are born. Preventing babies being born too early is a huge focus of research due to its massive impact upon baby's and families' lives and the NHS has specific targets to reduce how many babies are born early each year.

At the moment we assess pregnant women who we know have certain risk factors that could lead to preterm birth in our specialist preterm birth clinics. At these appointments the women have an internal ultrasound scan to measure the length of the neck of the womb (cervix) during the pregnancy. If the cervix is measured as short, additional medications or procedures can be offered to help reduce their risk of having a preterm birth. There is some evidence that a swab test taken from the vagina can help predict which women may deliver earlier and can be used alongside cervical length measurements to improve their risk assessment. Despite this service, there are still some women who will continue to have an early birth. Therefore, it is important to try and find new and better ways to find out which women might deliver earlier so we can offer the right treatment when needed.

We wish to use a new test which measures how "stiff" the cervix is as it may improve our current cervical length ultrasound test. If the cervix becomes softer too early during the pregnancy, this might be better at predicting if the baby will come too early compared to if the cervix was getting shorter. So far, this test has been used in non-pregnant women or pregnant women who don't have any risk factors for preterm birth. We want to see if those initial promising findings are also found in women who we know have risk factors for early birth and are therefore seen in our preterm birth clinic during pregnancy. By trying to identify a link between cervical stiffness and preterm birth, we hope to guide new targets for treatments to prevent preterm births in the future.

There are many other areas of interest in ongoing research into preterm birth including looking at microscopic organisms (such as bacteria, yeasts and viruses) found in the vagina during pregnancy that may help predict preterm birth. During this study we plan to collect two additional vaginal swab samples that can be used to explore this further in future research studies.

# Participant Information Sheet

**Why have I been chosen?**

We are inviting all women aged 18 years and older who attend the pre-term birth clinic to take part in this research study.

**Do I have to take part?**

No. It is up to you to decide whether or not to take part in this study. If you decide to take part - you will be asked to sign a consent form. You will be free to withdraw from the study at any time, without giving a reason. If you decide not to take part – this will not affect the care you or your family receives.

**What will happen to me if I take part?**

If you agree to take part in this study you will meet a member of the research team in the preterm birth clinic at your 16-week appointment. They will provide you with verbal and written information on the research study, answer any questions you may have and ask you to provide written consent for participation.

After you have given your consent to take part, they will arrange the following tests to be performed at your 16 , 20 and 24-week routine preterm birth clinic appointments. These tests include collection of a vaginal swab, a cervical stiffness assessment, and an internal scan of the cervix (we routinely offer this investigation at all preterm birth clinic appointments). At your first visit we will ask you to complete a short questionnaire outlining your experience of the tests during the clinic.

Further samples for use in future research, including two additional vaginal swabs, will be collected at your first appointment only. These swabs will only be used in future ethically approved research. Prior to taking these swabs you will need to complete a short questionnaire outlining some personal questions that some participants may mind intrusive. If you do not want to have these additional samples taken, you will not need to complete this questionnaire and you can still take part in this study.

All of these tests will happen during your routine clinic appointments, there will not be any additional visits to the hospital. Your clinic appointment will take longer and may last up to 45 minutes.

After your baby is born, we will collect information from your electronic medical records and your baby's electronic medical records regarding the events during and after your delivery. You will not be directly contacted at this time.

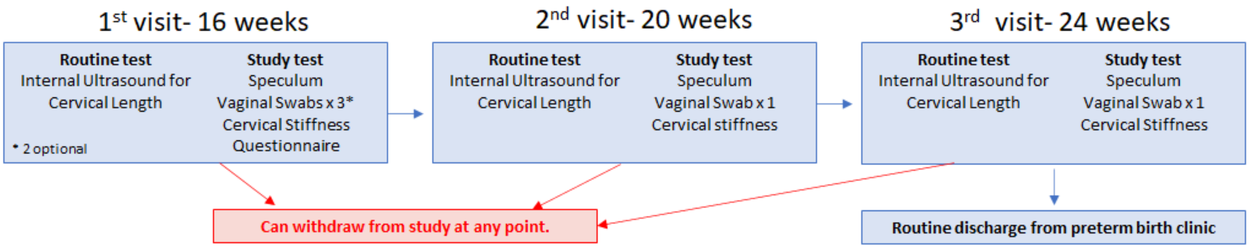

# Participant Information Sheet

**Will my taking part in this study be kept confidential?**

Yes. We will follow ethical and legal practice and all information will be handled in confidence. Any information you give us will be used by the research team in the course of the research. All samples and data will be stored securely. They will be coded, and no personal data will be available to the researchers. However, if any results provide clinically relevant information, we will inform your medical doctor.

We will also share the information collected during this study with other researchers, hospitals, universities, non-profit institutions and commercial companies in the UK, and worldwide, in a fully anonymised format (meaning those receiving the information will not be able to identify you). We will share fully anonymised findings from this study with the cervical stiffness device manufacturer, Pregnolia, in Switzerland.

**How will you use my data?**

We (study sponsor – the University of Liverpool) will need to use information from you and from your medical records for this research project.

This information will include your initials, name, date of birth, NHS number and contact details (address, telephone number and email). People will use this information to do the research or to check your records to make sure that the research is being done properly.

People who do not need to know who you are will not be able to see your name or contact details. Your data will have a code number instead.

We will keep all information about you safe and secure.

Once we have finished the study, we will keep some of the data so we can check the results. We will write our reports in a way that no-one can work out that you took part in the study.

What are my choices about how my information is used?

- You can stop being part of the study at any time, without giving a reason, but we will keep information about you that we already have.
- We need to manage your records in specific ways for the research to be reliable. This means that we won't be able to let you see or change the data we hold about you.
- If you agree to take part in this study, you will have the option to take part in future research using your data saved from this study.

Where can I find out more about how my information is used?
You can find out more about how we use your information

- at www.hra.nhs.uk/information-about-patients/
- our leaflet available from www.hra.nhs.uk/patientdataandresearch
- by asking one of the research team
- by sending an email to legalservices@liverpool.ac.uk, or
- by ringing us on 0151 795 0523.

# Participant Information Sheet

**What are the possible benefits of taking part?**

The results of this research will not be available in the course of your pregnancy and will not directly benefit you. However, we hope that the results of the study will enable us to improve future antenatal care provided to women who are known to be at risk of preterm birth by developing tests that will help us detect pregnancies at risk of preterm birth and guide future treatments to prevent preterm birth. We will ensure that your doctor is informed of any progress that means these new tests could be available for you in future pregnancies.

**What are the possible risks of taking part?**

The risks involved in this study have been carefully assessed and the main objective of the research team is to maintain yours and your baby's safety at all time. The additional study samples to be collected from you include vaginal swabs and bloods samples, and additional study procedures include an internal ultrasound scan and a cervical stiffness measurement.

- Vaginal swab collection will be performed using a speculum. A speculum is the same examination as when you attend for a smear appointment. Some women find this uncomfortable. It will be performed by an experienced practitioner and this examination will have no effect on your pregnancy.

- Cervical stiffness measurement will be performed at the same time as vaginal swab collection and would not cause any additional discomfort. A small device will be placed on the neck of the womb to create a small vacuum and gain three cervical stiffness measurements. Overall, the procedure will take no longer than a few minutes. It will be performed by an experienced practitioner with no risk to your pregnancy.

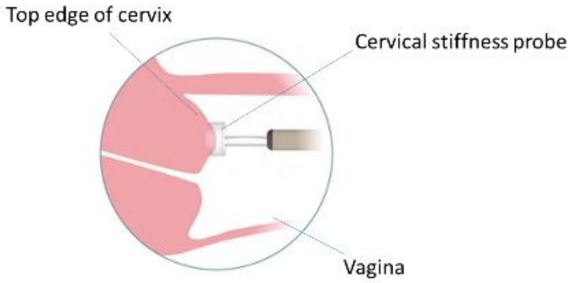


- Ultrasound has an excellent safety record and will not harm your baby.

**What will happen if I don't want to continue in the study?**

You are free to withdraw at any time throughout the course of the study, without explanation. The care you or your family receives will not be affected in anyway. If you withdraw from the study we will not take any further cervical stiffness measurements or collect any further vaginal swabs from you. If you wish, we will destroy any stored samples that were taken from you and no further information on how your pregnancy progressed will be collected for the study.

**What will happen to any samples I give?**

Vaginal swab samples collected specifically for this study will be coded and stored separately to any personal data (name, date of birth, contact details etc.) you provide to us. Throughout the course of the study your samples will be stored at secure laboratory facilities with restricted access and nobody outside of the study will have access to any of your confidential information

# Participant Information Sheet

With your permission, any vaginal samples taken for future research will be stored in the Harris Wellbeing Preterm Birth Centre laboratories, Centre for Women's Health Research, University of Liverpool for storage in accordance with The Human Tissue Act. We will ask if you would be willing to gift (donate) your stored samples to be used for other ethically approved research studies into pregnancy related problems in the future. These analyses may take place in hospitals, universities, non-profit institutions or commercial laboratories worldwide. The samples and data will be sent in a fully anonymised format meaning those performing the analyses will not be able to identify you

If you do not wish to take part in samples for future research you can still take part in this study. The consent form for this study allows you to indicate your preference.

**What will happen to the results of the research study?**

It is intended that once the study is complete the results will be published as research papers in medical journals. We will also engage with our patient support groups via social media to share our findings. No data will be published that will allow individuals to be identified.

**Where can I get further information or discuss any problems?**

Please visit https://en.pregnolia.com/ for further information regarding the cervical stiffness measurement device including patient experience testimonials and videos of the device in use.

If you have any questions or worries about any aspect of this study, please contact a member of the pre term birth team on 0151 702 4608. If your concerns are not resolved, you can contact the Patient Advisory Liaison Services (PALS) on 0151 702 4353. You can also visit PALS by asking at the hospital reception.

**Who is organising and funding the research?**

The University of Liverpool is the Sponsor of this research and is conducting this research study. The study is funded by The Harris-Wellbeing Preterm Birth Centre and is managed by the Centre for Women's Health Research, University of Liverpool.

**Who has reviewed the study?**

All research in the NHS is looked at by an independent group of people, called a Research Ethics Committee, to protect your interests. This study has been reviewed for ethical considerations and given a favourable opinion by members of the Wales REC 3 Research Ethics Committee.

**Contact for further information**

Should you have any further queries regarding this study, please contact:

Dr. Elizabeth Medford, Clinical Research Fellow, Harris Wellbeing Preterm Birth Centre, Centre for Women's Health Research, The University of Liverpool, Liverpool Women's Hospital, Crown Street, Liverpool, L8 7SS

Email: PRECISION@liverpool.ac.uk

# Participant Information Sheet

**Thank you for taking the time to read and consider this information sheet. Should you decide to take part in the study, you will be given a copy of the information sheet and a signed consent form to keep.**
