# Supplementary material for: The PRECISION study protocol: Can cervical stiffness in the second trimester predict preterm birth in high-risk singleton pregnancies? A feasibility, cohort study
Source: PLoS One. 2025 Feb 21;20(2):e0316297. doi: 10.1371/journal.pone.0316297 (PMC11844860; doi:10.1371/journal.pone.0316297)
Supplement: Attachment — Submitted filename: Response to Reviewers .docx [file pone.0316297.s004.docx]

Dear Academic Reviewers,

Thank you for taking the time to review my manuscript; “The PRECISION study protocol. Can cervical stiffness in the second trimester predict preterm birth in high-risk singleton pregnancies? An exploratory, prospective cohort study”.

I can infer from your review that that nature of the study as “exploratory” was not clear enough. Therefore, to make the aim of this exploratory study completely clear I have changed “exploratory” to “feasibility” and included clear feasibility outcomes to explain the purpose of this study.

I would like to highlight that reference to fetal fibronectin samples has been removed from this manuscript due to the recent statement by Hologic withdrawing this product from the market. Cervical stiffness assessments will now be compared with cervical length only, as this remains the only screening tool currently available in clinical practice.

Please find below our response to each of your individual points.

Major issues

1) The authors decided upon a patient recruitment target of n = 60 for the PRECISION study without a rationalization (sample size calculation) of this number in view of the study objective(s), i.e., the use of Cervical Stiffness (as assessed with the Pregnolia device) as a predictor for preterm birth in an (asymptomatic) high risk patient population. The authors justify their choice of not having performed a power analysis due to the Pregnolia device being a novel device with limited clinical data.
It is noted that Breuking et al recently published a study protocol which targets the same patient population and has largely the same aims as the PRECISION study [Cervical Stiffness as Assessment of cervical softening and the prediction of preterm birth (STIPP): protocol for a prospective cohort study https://bmjopen.bmj.com/content/bmjopen/13/11/e071597.full.pdf].
Different from the PRECISION protocol authors, Breuking et al provided a sample size estimation which suggests a minimal sample size of n=227 to evaluate Cervical Stiffness as a predictor for spontaneous preterm birth. Given the high degree of similarity in research question and research context on the one hand, yet the largely divergent recruitment targets, it is advised to verify (non-)futility of PRECISION by performing a formal sample size calculation or provision of another justification of the sample size. With Breuking et al in the public domain, the authors may want to replicate the power analysis as conducted by Breuking et al, using local estimates of prevalence etc, Based on their findings, the authors may possibly want to adjust the study aims accordingly as per the option to amend the protocol as per the language in the first paragraph of the “Introduction” on page 11 of the Precision Study Protocol.
In addition, given the apparent similarities between the PRECISION and STIPP (more specifically, the A-STIPP sub-study), the authors should reference Breuking et al and discuss similarities and differences between the studies.

**Author response: To provide a power calculation to assess if cervical stiffness is an appropriate tool for use in clinical practice several pieces of information are required, including a clear research question. Although the expected prevalence of sPTB <34 weeks is known from the high-risk population that is being sampled, it is not known what the prevalence of disease is amongst women consenting to inclusion. These women have all had a previous spontaneous preterm birth or late miscarriage and may not wish to have any invasive procedures, particularly involving or affecting the cervix. Breuking et al have used a standard level of variance for their calculation, however there is no clinical data yet available to show that the Pregnolia device can achieve this level of variance in this population. There is also an assumption that this tool will provide accurate readings in each recruited individual, despite never having been tested in this population. There may be a training period required for the user to get used to the equipment, resulting in unreliable readings. The manufacturers recommend taking triplicate measurements of cervical stiffness, but do not offer any guidance on which of these three measurements or how these measurements should be used in analysis. It is also unclear how one measurement will affect the following measurements given that the sampling point on the cervix is the same. We are performing a feasibility study recruiting over 15 months to address some of these questions and to determine if this is an intervention which is appropriate for further testing. Results will facilitate an informed sample size calculation for such a study and study design, if appropriate.**

**Our proposed figure of recruitment of 60 patients in our study period of 15 months was a conservative estimate based on our ability to recruit to other biomarker studies in the same population (ref Gupta JK, Care A, Goodfellow L, Alfirevic Z, Müller-Myhsok B, Alfirevic A. Genome and transcriptome profiling of spontaneous preterm birth phenotypes. Sci Rep. 2022 Jan 19;12(1):1003. doi: 10.1038/s41598-022-04881-0. Erratum in: Sci Rep. 2022 Feb 1;12(1):1986. doi: 10.1038/s41598-022-06338-w. PMID: 35046466; PMCID: PMC8770724.). Based upon the biomarkers study, our preterm birth rate in this population is estimated at 18% and therefore we might expect 10 preterm births in 60 recruits. If this number is achieved, we may be able to perform some basic prediction analysis of the test with logistic regression assessing just one predictive variable. However, this is not the purpose of this feasibility research study, and we recognise that overestimation in event rates are common when data is used in this way (ref Oliver CB, Strub L, Sunnen et al. Accuracy of Event Rates and Effect Size in Estimation in Major Cardiovascular Trials: A systematic Review. Jama Netw Open 2024; 7(4): e248818. Doi: 10.1001/jamanetworkopen.2024.8818)**

MINOR ISSUES

1. Abstract

1.1. Background
- To frame the PRECISION study, the authors identify that Cervical Length assessment, the best [current] available test for PTB prediction in a high-risk [pregnancy] population, has limited value in PTB risk prediction in the low-risk population. Given that the authors identify the lack of adequate PTB prediction in the low-risk population as the most pressing unmet clinical need, the background section would benefit of a justification/rationale why the results of the PRECISION study, as obtained in a high-risk population, would help in addressing the unmet clinical need in the low-risk population.
**Author response:**

**Predictive tools are affected by prevalence of disease. As the prevalence increases the PPV increases and the NPV decreases. In the low-risk population, the prevalence of preterm birth is naturally much lower. However, if this test shows no predictive value in the high-risk population, it almost certainly will not merit testing in the low-risk population.**

**Taking on board your comments we have amended the background to ensure the focus population of this feasibility study remains clear, the asymptomatic high-risk population.**

1.2. Methods

- Line 29: remove “non-invasive”
**Author response: Amendment made as suggested (line 40).**

1.3. Discussion
- Line 41: contrast cervical stiffness as a novel screening tool with the current screening tools (CL, fFN).
- Line 42: the authors may want to re-evaluate whether exploration of combining cervical stiffness, CL and fFN, into a multivariable model (cf. synergistic potential) is possible within the sample size.
**Author response; Amendments made to discussion to ensure clarity in feasibility nature of the study. All references to fFN have been removed due to the unexpected removal of the product from the market by Hologic.**

2. Introduction:
- Lines 50-59: In the first paragraph, the authors are providing some background to the PRECISION study. For the non-PTB researcher, the following would be helpful:
-reiteration of the clinical need **(amendments line 62-69**)
-the reasons why effective PTB risk prediction remains elusive **(amendments line 70-74)**
-mention of other predictors than cervical remodelling parameters like intrauterine infection and the role of FFN **(amendments line 75-79)**
-clarification that currently cervical remodelling assessment is based on sonographic assessments: CL (standard
care) and cervical consistency Index (subject to research) **(amendments line 76-81 & 84-89)**
-better explain why CCI has limitations (like?). It is noted that this statement appears at odds with the
statement made on page 13 of the Clinical Protocol, which mentions that CCI is an “easily, reproducible
assessment and effective in the prediction of spontaneous PTB”.**(amendments line 89-92 “application of these proposed cervical consistency assessment tools relies upon expert ultrasound skills using expensive resources and no objective technique to assess cervical softness during pregnancy has so far been well established in clinical practice**”**)**
- Line 66: The predictor under investigation is better defined as the Cervical Stiffness as obtained with the Pregnolia device and expressed as the CSI (mbar).**(amendment line 99)**
- Line 66: Within the context of PRECISION, it is unclear whether CSI is evaluated as a diagnostic biomarker (to confirm preterm labour; if yes – discussion should discuss comparison with QUIPP app decision support tool) or as a risk biomarker; it would be helpful if the authors can clarify this ambiguity, f.i, using the publicly available FDA-NIH resource on different types of biomarkers [https://www.ncbi.nlm.nih.gov/books/NBK338449/] **(amendment line 99- “risk biomarker”)**
- Lines 70-71: add reference to clinical guidance [NICE?] containing current gold standard practice as referred to **(reference added line 107)**
- Lines 70-73: The authors appear to suggest the use of longitudinal data for comparing CSI with CL and fFN; it is noted that such analyses are not mentioned in the statistical analysis? **(statistical analysis has been amended to address this and to reflect feasibility outcomes from this study)**
- Lines 73-74: As it stands, it is unclear which and how data from the PRECISION study will be used to inform a future clinical trial. It would be helpful if the authors can add this detail in the Discussion. **(amendment line 104-110 and further clarification in feasibility outcomes statistical analysis)**
- Note: The Background Section 1.1 in the actual Study Protocol is very well organized and clearly presented. The authors may consider replicating the same background presentation sequence (albeit condensed)

**Author response: Thank you for your comments. We have amended the introduction to give a full background to the non-PTB researcher as to the relevance and importance of this research.**

**We have addressed all the line suggestions as indicated above and made amendments accordingly.**

3. Materials & Methods

3.1. Study Design
- Line 79: The language suggests that recruitment occurs at first clinical appointment which happens between 14+0
– 25+6 weeks? Please clarify that recruitment is at first clinical appointment in the PTBC [14+0 – 17+6 weeks],
with 2 additional CS analyses scheduled as part of routine appointments scheduled before 25+6 weeks?
It is noted that the 3rd text box from the top in Figure 1 contains the same ambiguity.
- Line 84: If possible, reference guidance which outlines routine PTBC care
- Line 85: clarify that the questionnaire is only offered post first CL assessment.
**Author response: Patients can be recruited at any gestation in the PTBC from 14-25+6 depending on when they attend clinic. Patients who are recruited at study visit A will be between 14-17+6 weeks gestation, we would then hope to obtain further CS assessments at their subsequent clinic appointments that will correspond with Study visit B (18-21+6 weeks) and Study visit C (22+0-25+6). However, if a patient is first recruited when they attend clinic at 18+2 weeks, their study procedures at that visit will correspond to study visit B, they will have already missed study visit A.**

**This study design allows for patients who may miss their first appointment or who have been referred to clinic late and gives them the opportunity to take part in the study even if they miss time point study visit A. The ability to determine which study visit timepoint achieves the best recruitment will inform future study design.**

**Amendments made to figure 1.**

**All guidelines are referenced at the end of the paragraph on line 127.**

**We confirm that the questionnaire is only offered at the participants first study visit (can be A,B or C depending on their gestation). Figure 1 has been amended to make this clear.**

3.2. Study Objective
- Line 106: the language in objective 1.3 is unclear, can the authors clarify what is meant with “… explore the relative association with gestation at delivery”.
- Line 109: Objective 3 – details on how this study will inform a next study would be helpful; see higher.
**Author response: As a feasibility study, one of the aims is to search for any possible associations that may be worth following up in a subsequent larger study. (Thabane, L., Ma, J., Chu, R. *et al.* A tutorial on pilot studies: the what, why and how. *BMC Med Res Methodol* 10, 1 (2010).** [**https://doi.org/10.1186/1471-2288-10-1**](https://doi.org/10.1186/1471-2288-10-1)**). In this study we will be exploring whether cervical stiffness assessments are acceptable in this population, whether an appropriate recruitment rate can be achieved and evaluating the validity of the assessment tool when used in clinical practice. As part of our clinical outcome, we will explore whether cervical stiffness assessments in the second trimester have any association with the date at which delivery occurs, inferring if there is any possible relationship with preterm birth.**

**Further detail of how this feasibility study will inform future design has now been included in the introduction, study outcomes, statistical analysis and discussion.**

3.3 Inclusion and Exclusion Criteria
- OK

3.4. Sample Size Calculation
- Refer to Major Issue #1. It is noted that the authors have estimates on # referrals, # patients meeting eligibility, recruitment rate. If the authors can generate an estimate of the prevalence of sPTB in their Preterm Birth Clinic, they should be well equipped to conduct a power calculation. At a minimum, the latter prevalence estimate would allow for estimating the number of expected SPTB<34 weeks and application of the crude rule-of-thumb of having at least n=10 cases (or controls) per variable considered in a multivariable analysis.
**Author response: This section has now been moved to statistical analysis (line 270). Justification as to why we have not performed a sample size calculation for this feasibility study has been addressed in the response to Major concerns (above) and in the manuscript amendments (lines 272-287).**

3.5. Study Procedures

3.5.1. Pregnolia System
- OK

3.5.2. Cervical Stiffness Assessment
- Lines 175 – 177: Can the authors elaborate on how the clinician will be blinded to the CSI as the schematic in Figure 2 and the language (line 163) suggests that the CSI is displayed on the control unit when performing the measurements

**Author response: Line 190 explains that the measurement is audio-guided, therefore the clinician does not need to see the control box results to know the measurements have been taken, therefore can be blinded to the results. Amendment made to line 203 to ensure clarity.**

3.5.3. Cervical Length Assessment
- OK

3.5.4. Fetal Fibronectin Assessment
- Lines 193 - 200: Content of this first paragraph is better presented in the introduction
- Lines 198 – 200: Based on the outcome of above looked-for sample size calculation and /or estimation of sPTB <34 weeks expected, the authors are advised to re-evaluate whether this objective is meaningful within the n = 60 context of PRECISION

**Author response: As addressed earlier, this section has been removed from the manuscript due to the recent unexpected Hologic withdrawal of the product from the market.**

**Table 1 outlining Schedule of Study Procedures added into manuscript and removed from supporting information as per journal editor requirements (line 232).**

3.5.5. Post Assessment Questionnaire
- Lines 215 – 218: This content is more appropriately presented under Section Materials Methods – Study Design.

**Author response: Amendment made as suggested and moved to study design (lines 120-123)**

3.5.6. Study Outcome
-Lines 222 – 223: Use of “definitive” language can improve readability, e.g: The primary outcome is…. ; sPTB is defined as…
**Author response: Feasibility outcomes included to ensure full clarity of the nature and purpose of this exploratory study (lines 233-257). Amendment made to clinical outcome to improve readability as suggested (lines 259-265)**

3.5.7. Study Endpoint
- OK

3.5.8. Statistical Analysis
- This section would benefit from a more structured organisation, fi,
- Descriptive statistics will be generated and presented as means (SD), median (interquartile range [IQR]), and
frequency of observations (percentages), as appropriate. Comparisons of patient characteristics between
women with and without the primary outcome sPTB<34 weeks will be performed using chi-square
(categorical data) or student-t test or Mann−Whitney U tests, as appropriate following normality testing
(Shapiro-Wilk).
[Note, the study’s n is sufficient to perform chi-square tests, there is no need of Fisher’s exact test]
- If there are >= 10 events,… logistic regression and ROC cure analysis will be performed to evaluate the
prediction performance of the predictors CS, CL and FFN… ;
Note: meaningful cut-offs are typically informed by clinical utility and typically require for selecting a criterion,
fi. fixed False Positive Rate = XX%; given the exploratory nature of PRECISION, the authors may consider this
as an objective for a follow-up study.
- [Multivariable analysis will be performed using Log Reg …etc;
Note: The authors are advised to re-evaluate whether this analysis is meaningful within the n = 60 context of
PRECISION]
-A p<0,XX will be used as the significance level in all statistical analyses
- Note: In other places, the author appear to indicate that prediction analysis will involve the use of longitudinal data as well. If this is the case, then the authors should update the Statistical Analysis Section with a description of what prediction modelling methods will be applied.

**Author response: Amendments made to outline statistical plan for feasibility and clinical outcome data (lines 284-288 & 290-303).**

3.5.9. Data Management Plan
- With recruitment already started, use of definitive language may be more appropriate in this section

**Author response: Amendments made to definitive language as suggested.**

3.5.10. Consent and Criteria for Withdrawal
- OK

3.5.11. Monitoring and Safety
- With recruitment already started, use of definitive language may be more appropriate in this section
- Line 278: it may be appropriate to qualify the Pregnolia SAE reporting to e.g. a certain time point, like no SAE were reported at time of starting the PRECISION study. **(amendment line 335)**

**Author response: Amendments made to definitive language as suggested.**

3.5.12. Ethical considerations and Declaration
- OK

3.5.13. Status and timeline of the study
- OK, refer to Major Issue Section regarding (non-)futility of the PRECISION study

**Author response: Please note an extension to recruitment was made after the submission of the previous manuscript. Now updated to reflect true recruitment period for PRECISION. We hope our amendments and answers to the previous comments have addressed your concerns regarding futility of the PRECISION study.**

4. Discussion
- Lines 301-302: The authors suggest that the effect of PTB interventions on CSI will be captured. There is however no mention of this in the Study Objectives nor in the Statistical Analysis Section. It would be helpful to the reader to understand how the authors will assess such intervention effects as part of the Statistical Analysis. **Amendment made to remove from discussion section to ensure clarity.** **This data will be shown as descriptive statistics only, this has been outlined in the clinical outcomes (line 265).**
- Lines 303-307: The authors should add some commentary regarding the published study protocol of Breuking et al vs the PRECISION protocol. **We have amended the discussion to ensure there is no ambiguity and to reflect the aim for feasibility results from this study. As suggested by the reviewers we have addressed the published study protocol from Breuking et al who are aiming to complete a definitive study in comparison to our decision to undertake a feasibility study first (lines 353-263).**

**Author response:**

5. Author’s Contributions
- OK

**Author’s response: We have added S Lane as an author for the amended manuscript. (line 377)**

6. Acknowledgments

- It is noted that the funding by Pregnolia is not disclosed in the official Study Protocol; Section 7.9.

**Author’s response: PLOS ONE journal requirements have asked me to remove my funding statement from the manuscript as it is disclosed elsewhere in the submission form. Similarly, funding by Pregnolia has been disclosed through separate documentation to the study protocol to both the Sponsor and ethics committee.**

Thank you for taking the time to review our amended manuscript. I look forward to hearing the outcome of your further review.

Kind regards,
Elizabeth Medford
