# Supplementary material for: The PRECISION study protocol: Can cervical stiffness in the second trimester predict preterm birth in high-risk singleton pregnancies? A feasibility, cohort study
Source: PLoS One. 2025 Feb 21;20(2):e0316297. doi: 10.1371/journal.pone.0316297 (PMC11844860; doi:10.1371/journal.pone.0316297)
Supplement: Attachment — Submitted filename: Response to reviewers.docx [file pone.0316297.s005.docx]

Dear Academic Reviewers,

Thank you for your review of my revised manuscript. I have addressed your further suggestions as outlined below.

ADDRESSING THE MAJOR ISSUES IDENTIFIED

It is noted that in their response to the “Major Issue”, the authors justify the PRECISION feasibility study in the context of the larger Breuking study by highlighting the latter assumes a standard level of variance to inform their power calculation. This justification is conducive to the reviewer. However, variance analysis is not (explicitly) part of the statistical analysis plan. The authors are invited to amend their SAP accordingly.

At the same time, it is advised to briefly explain the appropriateness of Cronbach’s alpha (used to e.g. assess science education “instruments”) in the context of replicate measurements of a vacuum pressure to assess tissue stiffness. Classic estimations of variance (standard deviation, %CV) etc may be more appropriate. Typically, triplicate measurements are used to generate a more precise estimate (mean) given that each measuring procedure has some variance. [triplicate measurements are however not ideal to estimate the within-run imprecision directly]. In their response to the “Major Issue” and the SAP, the authors appear to suggest that there may be other (biological) effects at play (other than plain measurement imprecision); is this the reason why Cronbach’s alpha is proposed to gauge measurement reliability? This warrants some further explanation, e.g. in the discussion.

**Author response:**

**In this feasibility study we will obtain an estimate of the variability in the outcome measure which can then be included in a sample size calculation for the full definitive study. I have made an amendment to the statistical analysis plan to clarify this. Lines 299.**

**Triplicate measurements have been taken as per the manufacturer guidance in using the Pregnolia System. The cervical tissue has natural elasticity and takes up to 30mins to return to its normal tissue structure after 1 reading, therefore when obtaining 3 readings in quick succession (as instructed to do so) there is expected to be a difference in the stiffness readings obtained, the first being the highest with the most pressure required to deform the tissue by 4mm, and as the tissue will not yet have returned to its natural state the 2^nd^ and 3^rd^ readings will be expected to require less pressure each time. Despite being informed by the manufacturer to take 3 successive readings, they have not advised on how to interpret or use those readings for clinical practice. Determining the reliability of the device readings will inform how to interpret and use these results in real clinical practice. This is referred to in the Discussion (line 367) and with the amendments made to the SAP the use of Cronbach’s alpha to achieve this should be clearer (line 292).**

**According to our statistician, determining Cronbach’s Alpha assesses the reliability of the device to produce consistent readings at different time points. It compares shared variability, among the measurements with the overall variance. If the device is reliable then there should be high covariance amongst the measurements relative to the variance. Added to statistical analysis plan. (line 292).**

MINOR ISSUES
1. Abstract
1.1. Background
1.1.1. Line 29: Suggested rephrasing: “Therefore, existing care pathways for managing PTB risk can potentially benefit… “ **Line 29 Amended as suggested.**
1.2. Methods
1.2.1. Line 37: Suggestion to replace “All women” by “All study participants….” **Line 37 amended as suggested.**
1.2.2. Line 45: Suggestion to replace “This assessment….” By “The cervical stiffness index data will be…”**Line 45 amended as suggested.**
1.3. Discussion
1.3.1. Line 50: Suggested Rephrasing: This is an exploratory study to assess the use of…**Line 50 amended as suggested.**

2. Introduction:
2.1.1. Line 64: Suggested Rephrasing: Unfortunately, it remains…**Line 63 amended as suggested.**
2.1.2. Line 70: Suggested Rephrasing: Understanding the events leading to sPTB remains…**Line 70 amended as suggested.**
2.1.3. Line 74: Replace “researching” by “predicting” **Line 75 amended as suggested**.
2.1.4. Line 91: “…expensive resources. So far, no…”**Line 92 amended as suggested**.
2.1.5. Line 97: Remove “ongoing” **Line 98 amended as suggested**.
2.1.6. Line 101: Suggestion to Replace “explore” by “investigate” **Line 102 amended as suggested.**
2.1.7. Line 102: Suggestion to Replace “relevant” by “representative” & “ensure” by “justify a” **Line 103 amended as suggested.**
2.1.8. Line 103: Suggested Rephrasing: ….predictive study and inform its appropriate design and power. **line 104 amended as suggested**.
2.1.9. Line 104: Suggestion to Replace “explore if” by “confirm whether”. **Line 105 amended as suggested.**
2.1.10. Line 106: Add “also”: The study will also… **Line 107 amended as suggested.**
2.1.11. Line 109. Move/adopt the following language from Section “Statistical Analysis- sample size” to here: … in these patients. “ In addition, the impact of the introduction of the Saving Babies Lives Care Bundle v3 on patient participation and local PTB rates in the asymptomatic high PTB risk pregnancy population will be determined.” **Amendments made, but moved to study outcomes rather than introduction. Lines 235-248.**
3. Materials & Methods
3.1. Study Design
3.1.1. Line 117: Suggested Rephrasing: “…during their PTB surveillance at the…” **line 119 amended as suggested**
3.1.2. Line 118: Suggested Rephrasing: “The CSI measurements will be taken alongside…” If possible, reference guidance which outlines routine PTBC care **Line 130 amended as suggested and reference added.**
3.1.3. Line 123: Suggested to move/modify language lines 130-131 to Line 123, e.g.: “Participants attending at the Study Visit A time window will be invited to consent to gifting… etc” **Line 125 amended as suggested.**
3.1.4. Line 124: Suggestion to Replace “coordinate” by “coincide” **Amended as suggested, line 128.**
3.2. Study Objective
3.2.1. Line 146: Suggested Rephrasing: “Upon positive conclusion of the PRECISION feasibility study, to inform the design…” **Line 150 amended as suggested**.
3.3. Study Population
3.3.1. Line 151: Suggested Rephrasing: “The Precision study population… “ **Line** **155 amended as suggested**
3.3.2. Line 153: Replace “Patient’s fulfilling” by “Patients meeting” **line 157 amended as suggested**
3.4. Inclusion and Exclusion Criteria:
3.4.1. Line 169: Spell out “LLETZ” **line 173 amended as suggested**

4. Study Procedures
4.1. Pregnolia System
4.1.1. Line 191: Suggested Rephrasing: “The Pregnolia system provides audio guidance to the clinician by indicating….**Line 195 amended as suggested.**

4.1.2. Line 191: Add: clear “operation” instructions **Line 197 amended as suggested.**
4.2. Cervical Stiffness Assessment
4.2.1. OK
4.3. Cervical Length Assessment
4.3.1. OK
4.4. Table 1:
4.4.1. Remove the row with row label: “Fetal fibronectin swab collection” – unless these were collected in patients already enrolled. If so, add an asterisk with a brief note that this procedure was discontinued at Date following removal of fetal fibronectin test from UK market by manufacturer. **Amended as suggested**.

5. Study Outcome
5.1. Feasibility outcomes
5.1.1. Line 257: add the aim metric (80% of ????) as Triplicate measurement reliability is not a common metric in the context of taking repeat pressure measurements. **Many thanks for highlighting this error. Cronbach’s alpha metric greater than 0.8 is considered to have good consistency, and so amendment from 80% to 0.8, line 269.**
5.1.2. It is suggested to add explicit language regarding inferring inputs for power calculation of a future study and introduce a feasibility outcome. **Suggestion noted, added at line 270.**
5.1.2.1. regarding collecting of sPTB rates in the total study population (participating and non-participating PTBC patients) to inform sPTB rate post care bundle v3 implementation – remove the language from section Statistical Analysis – Sample Size. **Removed from sample size as suggested and added line 271.**
5.1.2.2. regarding CSI measurement variance in this population to inform power calculation; remove the language from section Statistical Analysis – Sample Size. **Removed from sample size and added as line 274.**
5.1.2.3. Regarding recruitment and participation rates to inform number of participants needed ; remove the language from section Statistical Analysis – Sample Size **Removed from sample size and added as line 275.**
5.2. Clinical Outcomes
5.2.1. Line 261: Suggested Rephrasing: “The primary clinical outcome is…”**line 279 amended as suggested**
5.2.2. Line 263: Spell out “PPROM” **abbreviation added at line 166**

6. Study Endpoint
6.1.1. OK

7. Statistical Analysis
7.1.1. Move lines 273-276 to precede subsection ”sample size” **Moved to study outcome section, line 235-238.**
7.2. Sample Size
7.2.1. Make paragraph more concise; some of “explanation” language can be moved to introduction see point 2.1.11. Suggested Rephrasing: “ From previous studies at this study site, an 18% PTB rate was found for the local asymptomatic high risk population; equally these studies yielded acceptable recruitment / participation rates. Following the recent introduction of the Saving Babies Lives Care Bundle v3, which changes clinical practice, these rates need to be re-established. In this feasibility study…” **Suggestions noted, and amendments made moving section to study design (line 144), study outcomes line (239-248) and feasibility outcomes (line 271).**
7.2.2. When the authors amend the Feasibility outcomes as per suggestion 5.1.2, then the lines 284-288 can be removed. **Removed as suggested.**
7.3. Statistical Analysis
7.3.1. Lines 297-303: Mention that CL comparator diagnostic performance data will also be generated. **Line 306 amended as suggested**

8. Data Management Plan
8.1. OK

9. Consent and criterial for withdrawal
9.1. OK

10. Monitoring and Safety
10.1. OK

11. Ethical Considerations and declarations
11.1. OK

12. Status and Timeline of the Study
12.1. OK

13. Discussion
13.1. Line 372 – 373: Consider Rephrasing the concluding words: “…vital change in such a devastating cohort of obstetrics” is an incorrect phrase? **Amendment made to line 380.**

Thank you for taking the time to review our amended manuscript. I look forward to hearing the outcome of your further review.

Kind regards,
Elizabeth Medford
